# Efficacy of Platelet-Rich Fibrin in Preserving Alveolar Ridge Volume and Reducing Postoperative Pain in Site Preservation of Post-Extracted Sockets

**DOI:** 10.3390/medicina60071067

**Published:** 2024-06-28

**Authors:** Mohammed Alasqah, Radwa Diaaeldin Alansary, Khalid Gufran

**Affiliations:** 1 Department of Preventive Dental Sciences, College of Dentistry, Prince Sattam bin Abdulaziz University, Alkharj 11942, Saudi Arabia; 2 College of Medicine and Dentistry, Riyadh Elm University, Riyadh 12734, Saudi Arabia; radwa.d.alansary@gmail.com

**Keywords:** alveolar ridge, platelet-rich fibrin, tooth extraction, healing socket

## Abstract

*Background and Objectives*: In socket preservation, the goal is to minimize bone resorption after tooth extraction to maintain the volume and contour of the alveolar ridge. The use of PRF in post extraction sites may reduce ridge resorption by encouraging the growth of new bone and acting as a scaffold for tissue. In addition, PRF may enhance healing and minimize postoperative pain. The aim of this study was to evaluate the effectiveness of platelet-rich fibrin (PRF) in maintaining the ridges’ dimensions at the extraction site, in the maxilla and mandible, as well as its impact on post-extraction discomfort. *Methods*: The study was conducted on 60 patients presenting for extraction of posterior teeth and was randomly divided into three groups: group I PRF (*n* = 20), group II PRF + collagen (*n* = 20), and group III control (*n* = 20). Sockets were filled with PRF (group I) and PRF + collagen (group II). At baseline and follow-up after 3 months, CBCT was used to assess the bone dimensions. The postoperative pain evaluations were performed at 24 h, 3 days, and 7 days after the tooth extraction. The pain rate was evaluated using a numerical rating scale from the British Pain Society. *Results*: The study examined the effects of platelet-rich fibrin (PRF) and PRF combined with collagen on the height and width of the ridges, as well as the pain experienced by the patients following alveolar ridge preservation surgery. ANOVA and *t*-tests were used to evaluate and compare the ridge dimensions. Comparing the results to the control group, there were no significant differences in the height or width of the ridges. However, both the PRF and PRF + Collaplug^®^ treatments effectively reduced the short-term postoperative pain. *Conclusions*: The study findings suggest that platelet-rich fibrin (PRF) and PRF combined with collagen do not exert significant effects on ridge width and height compared to the standard treatment following alveolar ridge preservation. However, it is noteworthy that both the PRF and PRF + collagen treatments demonstrated efficacy in reducing postoperative pain in the short term, offering a potential advantage over standard treatment protocols.

## 1. Introduction

Following tooth extraction, the bone socket resorbs in both height and width as a result of bone remodeling and natural healing. According to a review of the literature, the ridge width decreases by 29–63% and height decreases by 11–22% after tooth extraction; the fastest and most significant bone resorption was observed in the first three months [1].

Dental implants are a popular choice for post-extraction prosthetics due to their durability and natural appearance. However, successful implantation requires adequate bone support, emphasizing the importance of considering the bone quality and quantity in decision making. Dental implants are preferred when there is sufficient bone for optimal results. They provide a convenient and beneficial way to restore function and appearance after teeth loss, but must meet various clinical, biomechanical, and biological conditions for long-term success [2,3]. 

According to Iasella et al. (2003), 50–60% of the alveolar bone atrophies in the first three months after tooth extraction. These results emphasize how crucial the first few days after tooth extraction are for the alveolar bone’s continued healing and transformation. This resorption leads to dimensional alterations and changes in the alveolar ridge [4].

The severity of healing patterns can cause esthetic challenges and pose challenges for future prosthetic restorations. According to He L. et al. (2009), the healing process is the body’s natural defense mechanism to preserve tissue integrity and maintain the organism’s life [5].

The physiological repair of the post-extraction socket, as well as the complicated process of bone cell migration and maturation, lead to specific bone resorption and apposition [6]. In socket preservation, the goal is to minimize bone resorption after tooth extraction to maintain the volume and contour of the alveolar ridge. Ridge preservation techniques are crucial for future dental procedures like implant placement or prosthetic restoration. Dental implantation procedures such as socket preservation, guided bone regeneration, growth factor use, immediate implant placement, and proper surgical technique have been developed to prevent bone atrophy and maintain alveolar ridge dimensions after tooth extraction [7,8,9]. However, some bone grafting options require more time to heal before new bone is incorporated into the graft site [10].

Atraumatic extraction is a surgical technique that minimizes trauma to the surrounding tissues and preserves the socket for potential future replacement [11]. Introduced by Dohan et al., platelet-rich fibrin (PRF) is a platelet product that is rich in autogenous growth factors and can be used in maxillofacial surgery to promote healing. Prepared from 10 cc of the patient’s blood without the use of chemicals (such as bovine thrombin or anticoagulants), PRF is a 100% autologous substance that contains platelets, leukocytes, and cytokines inside a robust fibrin network. Growth factors in high concentrations are released gradually over a period of 7 to 14 days from this concentrate. Numerous studies have demonstrated that PRF can hasten epithelial closure following surgery, improve soft tissue healing, and lessen discomfort and edema [12].

It has been suggested to preserve sockets by utilizing biomaterials and autologous platelet concentrates, such as platelet-rich fibrin (PRF) and platelet-rich plasma (PRP) with growth factors [13].

Second-generation autologous growth factors, or PRFs, promote healing and have been linked to early and efficient bone volume percentage and substance organization. Furthermore, PRF is a platelet concentrate that contains leukocytes in a thick fibrin matrix. It may be easily made by centrifuging autogenous, non-anticoagulated blood [14].

Preservation techniques, including the use of PRF, aim to mitigate this resorption by providing a scaffold for tissue regeneration and promoting the formation of new bone. In addition, according to Buchwald et al., discomfort, edema, infections, dry socket, and trismus are possible post-extraction consequences of PRF and may contribute to reduced postoperative pain and improved healing outcomes [15,16].

In dentistry, maintaining the ridge following tooth extraction is crucial. Research is needed to develop new methods to preserve soft tissue and bone, as dimensional changes may result in the loss of critical components for future tooth replacement and ridge contour.

Nevertheless, there is limited information on two crucial components of the use of platelet-rich fibrin (PRF) in ridge preservation studies: the assessment of postoperative pain and the assessment of bone using three-dimensional computed tomography (CBCT) radiographs. Thus, the purpose of this study was to assess the effectiveness of platelet-rich fibrin (PRF) in maintaining the ridges’ dimensions at the extraction site, in the maxilla and mandible, as well as its impact on post-extraction discomfort. The null hypothesis of this study was that there is no significant difference between using platelet-rich fibrin (PRF) on the dimensional change rate or the reduction of postoperative pain at the extraction site.

## 2. Materials and Methods

The current prospective study was conducted in the College of Dentistry, Riyadh Elm University. The Institutional Review Board of Riyadh Elm University approved this research proposal under approval number “FPGRP/2023/719/903/857”. Moreover, the study was conducted according to the guidelines of the Declaration of Helsinki (2013).

Patients were given a full explanation of the procedure’s advantages and risks. Every patient signed a consent form before participating in the clinical research. The sample size was calculated in G* power software (version 3.1) considering an 80% statistical power, 0.05 alpha, and 1.2 effect size, resulting in 19 samples per group for the current study [17]. However, to compensate for dropouts, we used 20 samples per group for the current study. A convenience sampling method was applied. A total of 60 patients whose posterior teeth indicated extraction due to non-restoration were included. The patients were randomly divided into three groups, each with 20 patients. The first group was the PRF group (test group, *n* = 20): patients had teeth extracted, and PRF was used alone. The second group, PRF + collagen (test group, *n* = 20), had teeth extracted and PRF was used along with Collaplug^®^,(Collagen Matrix, Inc., Oakland, NJ, USA) and for the third group (control group, *n* = 20), patients had their teeth extracted with no other treatment as a control group.

Patients were selected according to the following inclusion and exclusion criteria. Systemically healthy patients who are aged between 18 and 60 years with indications for the extraction of posterior teeth due to non-restorable reasons were included in the study. The study excluded pregnant women, smokers, patients with allergies, patients undergoing chemotherapy and radiotherapy, and patients with significant periapical lesions and periodontally compromised teeth. All patients underwent a detailed history-taking process before the surgical procedure. We conducted an intraoral examination of the patients to evaluate the condition of the tooth prior to extraction, and we also took a CBCT radiograph. Pain was evaluated using the numerical pain rating scale (NPRS), with a score range between 0, representing that there is no pain, and 10, representing extreme pain.

### 2.1. Surgical Procedures

Atraumatic extraction: Atraumatic tooth extraction was performed under local anesthesia using lidocaine 2% with 1:100000 epinephrine. Atraumatic extraction was performed with a PEREUR6 periotome (Hu-Friedy Mfg. Co., LLC, Chicago, IL, USA). After removing the tooth, we carefully cleared the extraction socket of granulation tissue using bone curettes and sterile saline. Evaluation of the bone, especially buccal bone, at the extraction site was performed gently using a periodontal probe.

PRF preparation: The patient provided 9–10 mL of blood. After blood collection, we placed the blood in plain tubes, added no additional anti-coagulant, and immediately centrifuged each blood sample for 12 min at 2700 rpm (400× *g*). (Hermle Labortechnik, Wehingen, Germany). As a result, three layers formed in the tube. The top layer is acellular plasma/platelet-poor plasma (PPP), the bottom layer is RBC concentrates, and the center layer is a fibrin clot/PRF. The fibrin clot was retrieved by using a sterile surgical tweezer, pulled out, and any RBCs attached to it were removed. It was then placed in the small cylinder of a PRF metal box. A piston was used to compress the clot carefully to obtain a plug form of the PRF.

In the first group, we placed the PRF at the extraction site and performed figure eight sutures. In the second group, we used the PRF, placed a collagen plug (Collaplug^®^) above it, and performed a figure eight suture. For the third group, the control group received only the atraumatic extraction. After the tooth extraction, a postoperative pain assessment was performed 24 h, 3 days, and 7 days later. A British Pain Society numerical rating scale was used to assess the pain level. The patient had follow-up appointments after seven days, one month, and three months. At the three-month follow-up, a second CBCT examination was performed.

### 2.2. Statistical Analysis

The Shapiro–Wilk test was used to determine the normality of the study’s variables. Variables such as pain were calculated using descriptive statistics. An independent *t*-test and ANOVA were applied to the data. SPSS version 22 (IBM-SPSS Inc., Armonk, NY, USA) was used for the analysis of the data, and significant differences were defined as *p*-values under 0.05.

## 3. Results

Table 1 presents data comparing the ridge width before and after treatment in the three groups: the control group, the platelet-rich fibrin (PRF) group, and the PRF combined with collagen group. The variables measured were the ridge width before treatment (mean) and after treatment (mean) along with the corresponding standard deviations (SD) for each group.

The table indicates that there were no statistically significant differences in ridge width before and after treatment among the three groups, as indicated by all the *p*-values being >0.05.

Table 2 presents a comparison of the ridge height before and after treatment among three groups: the control group, PRF group, and PRF + collagen group. For each group, the mean ridge height before and after treatment is provided along with the corresponding standard deviation (SD). Additionally, the table includes the *p*-values resulting from the *t*-test analysis, indicating the statistical significance of the changes observed in ridge height before and after treatment within each group.

Overall, the analysis suggests that there was no significant difference in ridge height after treatment compared to before treatment within each group, as indicated by the non-significant *p*-values.

Table 3 illustrates the pain scores reported by the participants in the three different groups (control group, PRF group, and PRF+ collagen group) over the course of one week. The pain scores were measured at three different time intervals: 24 h, 3 days, and 1 week post-treatment. For each time interval, the table provides the mean pain score along with the standard deviation (SD) for each group.

Across all groups, the mean pain score decreased progressively over time, indicating a reduction in pain levels as time elapsed post-treatment. Notably, the control group initially reported the highest mean pain score at 24 h compared to the PRF and PRF + collagen groups. However, by 3 days and 1 week post-treatment, both the PRF and PRF + collagen groups reported lower mean pain scores compared to the control group.

Overall, the results suggest that both the PRF and PRF + collagen treatments were associated with lower pain levels compared to the control group, particularly within the first 3 days post-treatment. However, the differences in pain scores among the groups became less pronounced by 1 week post-treatment.

## 4. Discussion

This study investigated the effects of platelet-rich fibrin (PRF) and PRF combined with collagen on ridge width, ridge height, and postoperative pain in patients undergoing alveolar ridge preservation following tooth extraction. The outcomes were compared to those of a control group receiving the standard treatment. The data comparing the ridge width before and after treatment are shown in Table 1 for three groups: the PRF (platelet-rich fibrin), PRF + collagen, and control groups. 

In the PRF group, the ridge width before treatment was 11.09 (SD 0.615), and after treatment, it was 10.98 (SD 0.589). The *p*-value for this comparison was >0.05. In the PRF + collagen group, the ridge width before treatment was 10.90 (SD 1.024), and after treatment, it was 10.84 (SD 1.031). The *p*-value for this comparison was >0.05. Table 1 indicates that there were no statistically significant differences in ridge width before and after treatment among the three groups, as indicated by all the *p*-values being >0.05. 

Consistent with our study, in a past study, the ridge width exhibited a smaller difference from baseline to 180 days in the PRF group compared to the control group (T: 0.75 ± 0.49 vs. C: 1.36 ± 0.70; *p* = 0.005), indicating a significant reduction in width variation in the PRF group [18].

The previous studies assessed the ridge contour at 5 mm from the crest and observed a considerably more significant change in width with the platelet-rich fibrin treatment four weeks after extraction. This was in contrast to self-healing sockets [19,20,21].

Few studies that used CBCT to quantify bone width were included in the meta-analysis, in contrast to the measurement of contour changes. The platelet-rich fibrin-treated group showed reduced bone width resorption in comparison to the natural socket healing group; nevertheless, no statistically significant difference was found. However, this comparison revealed substantial variability [22,23].

The primary objective of alveolar ridge preservation is to maintain the integrity of both the hard and soft tissues following tooth extraction, with the aim of facilitating optimal implant positioning for prosthetic purposes. The choice of preservation technique depends on the patient’s condition, the extracted tooth’s location, and the clinician’s preferences. Techniques like socket preservation using grafts and rapid implant placements reduce post-extraction bone loss and provide predictable implant placements [8]. Two studies investigated the dimensional changes of the alveolar process using PRF alone [19,23]. Both studies found that PRF significantly reduces the resorption of alveolar width from 8 weeks to as long as 6 months post-surgery. Notably, both studies utilized multiple PRF clots or membranes, with Alzahrani et al. [19] employing two membranes and Temmerman et al. [23] using three to seven membranes. The quantity of clots or membranes and the corresponding blood volume could influence the clinical outcome by potentially modulating the cellular environment within the socket [24].

Furthermore, they only employed a single clot compared to the multiple clots/membranes used by others [19]. Marenzi et al. observed improved soft tissue healing at 7, 14, and 21 days; however, interpreting these findings clinically is challenging due to the various healing indexes present in the literature [25]. 

According to Carmagnola et al. [26], there was a higher decline in bone width and height in the absence of any alveolar preservation procedures. 

Our research also compared spontaneously healed sockets filled with PRF + Collaplug and found comparable effects to those of Iasella et al. [4]. According to this study, using PRF + Collaplug essentially aids in ridge preservation and increases the bone density. 

Table 3 illustrates the pain scores reported by the participants in the three different groups (control group, PRF group, and PRF + collagen group) over the course of one week. The pain scores were measured at three different time intervals: 24 h, 3 days, and 1 week post-treatment. For each time interval, the table provides the mean pain score along with the standard deviation (SD) for each group. Across all groups, the mean pain score decreased progressively over time, indicating a reduction in pain levels as time elapsed post-treatment. Notably, the control group initially reported the highest mean pain score at 24 h compared to the PRF and PRF + collagen groups. However, by 3 days and 1 week post-treatment, both the PRF and PRF + collagen groups reported lower mean pain scores compared to the control group. Therefore, the current study partially rejects the null hypothesis of the study as there was a significant difference between the groups using platelet-rich fibrin (PRF) and the control group in reducing postoperative pain at the extraction site.

The *p*-values provided in Table 3 represent the statistical significance of the observed differences in pain scores among the three groups at each time interval, as determined by one-way ANOVA. The *p*-values for the pain scores at 24 h and 3 days post-treatment were statistically significant (*p* < 0.05), indicating that there were significant differences in pain scores among the groups during these time periods. However, at 1 week post-treatment, the *p*-value was 0.374, suggesting that there were no significant differences in pain scores among the groups at this time interval. 

Patients report pain as an important measure [27]. Two studies utilized the visual analog scale to gauge patient-reported outcomes [23,25]. Temmerman et al. [23] concluded that PRF notably alleviated pain sensations within 3 to 5 days, while Marenzi et al. [25] observed reduced pain in the PRF group for up to 21 days. However, neither study specified whether patients received adequate blinding. There are a plethora of studies investigating the effect of PRF on mandibular third molar extraction pain [28]. 

At all times, the patients in the PRF group reported much lower pain ratings. When comparing the control group to the PRF group on days 7 and 14, the VAS score values were lower in the former. According to Girish Kumar et al. [29], the PRF group’s postoperative analgesic consumption was considerably lower than that of the control group on the first and third days. 

In addition to a significantly lower peak pain level on the operation day in the PRF group compared to control group, lower pain levels on the 10th day were noted clearly in the PRF group in comparison to control group. Both groups’ VAS ratings decreased from baseline to the tenth day. The VAS values of the PRF group were consistently lower than those of the control group across all periods. The PRF group’s pain ratings returned to normal levels sooner than those of the control group [29]. 

The mean pain score for the PRF group was significantly lower at 2.90 (SD 0.96) after 24 h. It dropped to 0.550 (SD 0.60) after three days and stayed at this level for one week. Similar to the control group, the 3-day and 1-week periods’ *p*-values were 0.000, showing a substantial decrease in pain from baseline. 

In the study conducted by Carmagnola et al. [26], the control group also experienced a reduction in bone height of 2.12 ± 0.69 mm and a loss of bone width of approximately 1.71 ± 0.49 mm. 

The literature findings imply that PRF offers benefits in human alveolar ridge preservation due to its ease of use and straightforward handling. Histological examination of the newly formed bone confirmed that PRF enhanced both the quality and rate of bone formation, although its effect on reducing alveolar bone resorption in the extraction socket alone was not statistically significant [30]. 

The previous research compared the bone density, width, and height of three groups, as well as their soft tissue healing. Compared to the controls (group I), the PRF (group II) and PRF + Collaplug (group III) groups both demonstrated improved healing on postoperative day 7. Through releasing inflammatory cytokines and growth factors, PRF contributes to the repair of soft tissues. The preservation of bone height at four months postoperatively was similar in the PRF and PRF + Collaplug groups due to PRF’s role in stimulating the neovascularization of bone tissue, boosting new bone production, and maintaining bone height. The preservation of bone width was superior in the fourth postoperative month for the PRF + Collaplug group. When combined with PRF, the resorbable Collaplug^®^ plays a crucial role in socket preservation by stabilizing the initial clot, inhibiting the ingrowth of surrounding soft tissue into the socket during routine healing, and preserving the socket width after surgery [17].

### Strengths and Limitations

While the study provides valuable insights into the immediate outcomes of PRF and PRF + Collagen interventions, longitudinal follow-up studies are warranted to assess their long-term effects on ridge morphology and clinical outcomes such as implant success rates and patient satisfaction. Additionally, exploring variations in treatment protocols, such as different concentrations or combinations of biomaterials, may offer further insights into optimizing alveolar ridge preservation techniques. Overall, this research contributes to the growing body of literature on dental biomaterials and highlights the need for comprehensive assessment strategies to evaluate the multifaceted outcomes of alveolar ridge preservation interventions in clinical practice. 

Though many studies validate the influence of PRF on alveolar ridge preservation, some studies oppose the idea as no significant influence was observed. Therefore, further studies on PRF should be conducted for a better understanding of the clinical efficacy of PRF in extraction socket preservation and the consequent effects on post-surgical morbidity.

## 5. Conclusions

In conclusion, the study findings suggest that platelet-rich fibrin (PRF) and PRF combined with collagen do not exert significant effects on ridge width and height compared to the standard treatment following alveolar ridge preservation. However, it is noteworthy that both the PRF and PRF + collagen treatments demonstrated efficacy in reducing postoperative pain in the short term, offering a potential advantage over standard treatment protocols. 

## Figures and Tables

**Table 1:** Before and after width comparison among control, PRF, and PRF + collagen groups using *t*-test.

Group	Ridge Width Before	Ridge Width After	*p*-Value
Control group	10.89 (SD 0.567)	10.80 (SD 0.606)	0.673
PRF group	11.09 (SD 0.615)	10.98 (SD 1.031)	0.699
PRF + collagen group	10.90 (SD 1.024)	10.84 (SD 1.031)	0.843

PRF: Platelet rich fibrin; SD: Standard deviation.

**Table 2:** Before and after height comparison among control, PRF, and PRF + collagen groups using *t*-test.

Group	Ridge Height Before	Ridge Height After	*p*-Value
Control group	10.52 (SD 1.950)	10.42 (SD 1.883)	0.877
PRF group	10.02 (SD 1.040)	9.82 (SD 1.020)	0.595
PRF + collagen group	10.52 (SD 1.043)	10.27 (SD 1.017)	0.534

PRF: platelet-rich fibrin; SD: standard deviation.

**Table 3:** Pain scores among control, PRF, and PRF + collagen groups over 1 week using ANOVA.

Group	Pain at 24 h (Mean)	Pain at 3 Days (Mean)	Pain at 1 Week (Mean)	*p*-Value
Control group	5.25 (SD 1.91)	1.80 (SD 1.23)	0.100 (SD 0.44)	0.000
PRF group	2.90 (SD 0.96)	0.550 (SD 0.60)	0.000 (SD 0.00)	0.000
PRF + collagen group	2.70 (SD 0.92)	0.550 (SD 0.75)	0.000 (SD 0.00)	0.000
*p*-value	0.000	0.000	0.374	

PRF: platelet-rich fibrin; SD: standard deviation.

## Data Availability

All the data are contained within the article.
